# A Comparative Study of the Phenolic and Technological Maturities of Red Grapes Grown in Lebanon

**DOI:** 10.3390/antiox6010008

**Published:** 2017-01-26

**Authors:** Hiba N. Rajha, Nada El Darra, Sally El Kantar, Zeina Hobaika, Nicolas Louka, Richard G. Maroun

**Affiliations:** 1 Unité de Recherche Technologies et Valorisation Agro-alimentaire, Centre d’Analyses et de Recherche, Faculté des Sciences, Université Saint-Joseph, B.P. 11-514 Riad El Solh, Beirut 1107 2050, Lebanon; hiba.rajha@usj.edu.lb (H.N.R.); sally.kantar@net.usj.edu.lb (S.E.K.); zeina.hobaika@usj.edu.lb (Z.H.); nicolas.louka@usj.edu.lb (N.L.); 2 Faculty of Heath Sciences, Beirut Arab University, Tarik El Jedidah–Beirut, P.O. Box 115020 Riad EL Solh, Beirut 1107 2809, Lebanon; n.aldarra@bau.edu.lb

**Keywords:** glories, grapes, ITV, phenolic maturity, technological maturity

## Abstract

Grape harvest date is determined according to the technological and phenolic maturities. These parameters were calculated for different red grape (*Vitis vinifera* L.) varieties (Cabernet Sauvignon, Merlot, Syrah, Cabernet Franc) over four years (2008, 2009, 2010, and 2011) (642 samples). Titratable acidity and sugar content of the grapes were used to determine the technological maturity, whereas Glories (1 and 2) and ITV (Institut Technique de la Vigne et du Vin) methods were used to monitor their phenolic maturity. The ITV method allows the monitoring of phenolic maturity by the quantification of total polyphenol index and anthocyanins, while the Glories method enables the quantitative evolution of extractable anthocyanins and tannins of the grapes. A correlation was shown between the harvest dates obtained by both ITV and Glories (R^2^ = 0.7 – 0.93). Phenolic maturity of grapes can, therefore, be optimized by the application of both ITV and Glories. Similarly, a correlation was observed between technological and phenolic harvest dates. The effect of climate on the phenolic content of grapes was also studied. The highest temperatures (up to 25 °C) accompanied by the lowest rainfall (null value), induced the maximal concentration of polyphenols in grapes. Thermal and water stresses were also shown to enhance the grapes’ polyphenolic production.

## 1. Introduction

In the winemaking industry, wine quality is largely influenced by grape harvest time, which is defined by many parameters such as the technological and phenolic maturity of the grapes. Technological maturity is characterized by the sugar content, which determines the potential alcohol content of the future wine, as well as the titratable acidity and pH of grapes must, which in turn contributes to the color and quality of the wine. In wineries, the sugar content is usually determined by a refractometer to measure the refractive index or by total soluble solids (Degrees Brix) using density studies. Total titratable acidity and pH are respectively measured by volumetric titration and pH meter. Phenolic maturity, on the other hand, is evaluated by the ripeness stage of the skin, pulp, and seeds, thus facilitating the choice of the harvest date [1,2]. The concentrations of anthocyanins and tannins, which are the most abundant polyphenols in red grapes, are good indicators of phenolic maturity since they accumulate in the grape skins during the ripening process. Located in cell vacuoles, anthocyanins are easily released in the extraction medium when those vesicles are weakened by grape ripening. In wine, instability and reactivity of anthocyanins, together with co-pigmentation reactions, are responsible for the color changing of the wine [3].

Therefore, grape maturity is also defined by the extractability of polyphenols during the winemaking process [4]. Contrarily to the increase in anthocyanin and tannin concentrations in grape skins, seed tannins are less liberated during grape maturity [5]. Wine quality is also related to the climate, rainfall, grape cultivar and soil quality, which affect the phenolic content and grape ripeness [6].

In this study, the phenolic maturity of red grapes grown in the vineyards of Château KSARA S.A.L (Zahlé, Bekaa, Lebanon) was conducted by means of Glories [5,7] and ITV (Institut Technique de la Vigne et du Vin, Narbonne, France) methods [8]. The correlation between both methods was studied in terms of the resulting phenolic harvest dates, based on the extraction kinetics of different components such as anthocyanin content, total polyphenol index, and seed maturity. A comparison between phenolic and technological harvest dates was also undertaken. In addition, the effect of climate (rainfall, temperature) on the grape phenolic content was monitored over a period of four years (2008–2011). This data collection is the first of its kind in the Mediterranean region. It creates a reference about the maturity of the grapes grown in Lebanon over an extended period.

## 2. Materials and Methods

### 2.1. Raw Material

Different grape (*Vitis vinifera* L.) varieties (Cabernet Sauvignon (CS), Merlot, Syrah, and Cabernet Franc) from different vineyard parcels were studied over four years (2008, 2009, 2010, and 2011). Grape samples were received weekly for several months (from August to October) to monitor phenolic and technological maturity over grape harvest time. Samples were provided by Château KSARA (Zahlé, Bekaa, Lebanon) and identified by a code determined by the vineyard parcel name followed by the grape variety and the planting time of the vine. For example: a sample from domain ITANY, and a Cabernet Sauvignon variety planted in January 1994-1 is coded ITCS41. During the four years, 642 samples were analyzed. In this study, data for Cabernet Sauvignon vines were shown from different parcels and were coded as follows: MVCS51 (domain MANSOURA, Cabernet Sauvignon 1995-1), MVCSK0 (domain MANSOURA, Cabernet Sauvignon 1990), ITCS41 (domain ITANY, Cabernet Sauvignon 1994-1), ITCS42 domain ITANY, Cabernet Sauvignon 1994-2), ITCS51 (domain ITANY, Cabernet Sauvignon 1995-1), ITCS52 (domain ITANY, Cabernet Sauvignon 1995-2), TACS6 (domain TANAYEL, Cabernet Sauvignon B6), KACS31 (domain KANAFAR, Cabernet Sauvignon 1), and KACS32 (domain KANAFAR, Cabernet Sauvignon 2).

### 2.2. Technological Maturity

Technological maturity was determined by the sugar content, titratable acidity and pH. The sugar content was measured by the Brix degree through a digital refractometer (PR-101, Atago, Bellevue, WA, USA) (at 20 °C) and converted to sugar content (g/L) according to the ITV database [9].

Titratable acidity was measured by the acid/base titration using NaOH 0.1 N and bromothymol blue (4g/L) as an indicator dye. The pH of grape juice was measured using a pH meter (Consor C931, Bioblock Scientific, Paris, France) at 20 °C.

### 2.3. Sample Preparation for ITV and Glories Methods

Several bunches of grapes were received in plastic bags and 200 grape berries were randomly collected from each sample and ground for two minutes. Grape bunch samples were received from the winemaking company which marked four rows per plot for each vine variety. These rows were symmetrically chosen with regards to the size of the plot. Three vines were numbered per row and grape bunches were collected over the two arms of each of the 12 vines.

Fifty grams of the resulting grape juice were introduced in a 250 mL Erlenmeyer flask to apply the ITV method. Another 100 g were placed in two Erlenmeyer flasks (50 g of sample in each) to apply the Glories method.

#### 2.3.1. ITV (Institut Technique de la Vigne et du Vin) Method

The ITV reference method was used to measure anthocyanins and total phenolic compound contents of grapes during maturation [8]. Fifteen milliliters of ethanol (95%) and 85 mL of 0.1% HCl were added to the 50 g of grape juice. After 1 h of maceration at 20 °C, the sample was filtered through glass wool. A first sample dilution to 1/100 was done in distilled water and the absorbance was measured using a UV-VIS spectrometer (UV-9200, BioTECH Engineering Management Co. Ltd., Nicosia, Cyprus) at an OD (optical density) of 280 nm against a blank of distilled water. Total polyphenol index (TPI) was calculated as follows [10]:

(1) $$Total\;polyphenol\;index=OD_{280}\times 100\times \frac{\left(weight\;of\;grape\;juice+100\right)}{\left(weight\;of\;grape\;juice\right)}$$

A second sample diluted to 1/20 was done in 1% HCl and the OD was measured at 520 nm against a blank of distilled water. The concentration of anthocyanins (ANT) and the total anthocyanin potential (TAP) were estimated as follows:

(2) $$Anthocyanins\;\left(\frac{mg}{L}\right)=OD_{520}\times 22.75\times 20$$

(3) $$Total\;anthocyanins\;potential\left(\frac{mg}{kg}\right)=Anthocyanins\left(\frac{mg}{L}\right)\times 100\times \frac{\left(weight\;of\;grape\;juice+100\right)}{\left(weight\;of\;grape\;juice\right)}$$

#### 2.3.2. Glories Method

Fifty milliliters of aqueous solution at pH 3.2 were added to the first 50 g of sample. The pH 3.2 solution was prepared by adding 5 g of tartaric acid to water (1 L) with a pH adjustment to 3.2 by NaOH. Fifty milliliters of aqueous solution pH 1 (37% HCl in distilled water with pH adjusted to 1) were added to the second 50 g of sample. Samples were macerated for 4 h at 20 °C then filtered through glass wool. Anthocyanins and total phenolic contents were estimated.

The dosage of anthocyanins is based on the principle of anthocyanin discoloration by SO_2_ [11]. One milliliter of each filtrate (pH 1 or pH 3.2) was added to 1 mL of ethanol 0.1% HCl and 20 mL of concentrated 2% HCl. 10 mL of the mixture and 4 mL of distilled water were introduced in a first tube while 10 mL of the mixture and 4 mL of sodium bisulfite (15%) were introduced in the second tube. Bleaching is practically instantaneous. After 20 min, the optical density at 520 nm was measured against distilled water for both tubes. Anthocyanin concentration (*Ant*) was given in milligrams (mg) of anthocyanins per liter (L) and calculated as follows:

(4) $$Ant\left(\frac{mg}{L}\right)=875\;\times \left(OD_{tube\;1\;in\;water}-OD_{tube\;2\;in\;bisulfite}\right)$$

with 875 being the slope of the calibration curve obtained from malvidin-3-glucoside.

Following this calculation, two values are calculated as *Ant*_1_ and *Ant*_2_. From these values, several results will be provided:

The potential of easily extractable anthocyanins was calculated as follows:

(5) $$AntpH_{3.2}=Dilution\;factor\times A_{2}$$

The total anthocyanin potential was calculated as follows:

(6) $$AntpH_{1}=Dilution\;factor\times A_{1}$$

The percentage of extractable anthocyanins (PEA) was calculated as follows:

(7) $$PAE=\frac{A_{pH\;3.2}}{A_{pH\;1}}\times 100$$

Anthocyanin extractability (AE) or cell maturity index was calculated as follows [4]:

(8) $$AE=\frac{A_{pH\;1}\;-\;A_{pH\;3.2}}{A_{pH\;1}}\times 100$$

To estimate total phenolic richness (*TPR*) in the extracts macerated at pH 3.2, a dilution to 1/100 was conducted and the optical density was measured at 280 nm against distilled water. Then overall estimation of total phenolic compounds was calculated:

Total phenolic richness was calculated as follows:

(9) $$TPR=2\times OD_{280}\times 100$$

Seed tannins (*ST*) were calculated as follows:

(10) $$ST=RPT-skin\;polyphenols=RPT-\;\frac{ApH3.2\times 40}{1000}$$

The phenolic maturity of the seeds (*SM*) was calculated as follows:

(11) $$SM=\frac{ST}{RPT}\times 100$$

### 2.4. Statistical Analysis

All experiments were repeated at least three times (four measurements were done per sample (*n* = 4)). Average and standard deviations of the data were calculated. The average comparison test and the Fisher test (LSD) were applied to compare the results. The confidence interval was set at 5%. For statistical tests, the Statgraphics Plus 5.1 software (Statpoint Technologies, Inc., Warrenton, VA, USA) was used. Modeling of the experimental curves obtained was carried out using the Table Curve software (2D Windows version 2.03, San Rafael, CA, USA).

## 3. Results and Discussion

### 3.1. Harvest Dates of Different Cabernet Sauvignon (CS) Plots

**Figure 1:**
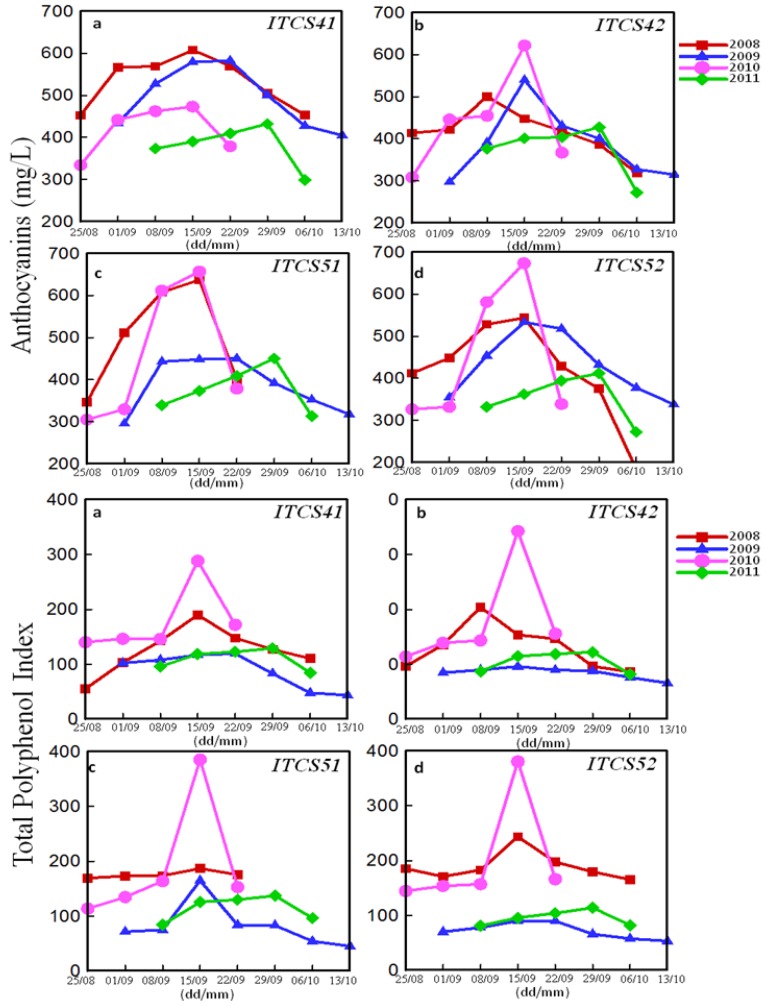
Anthocyanin content (mg/L) and total polyphenol index (IPT) (calculated by Institut Technique de la Vigne et du Vin (ITV) method) of four plots ITCS41 (**a**); ITCS42 (**b**); ITCS51 (**c**); and ITCS52 (**d**) of Cabernet Sauvignon grapes as a function of time (day (dd)/month (mm)) over four years (2008, 2009, 2010, and 2011).

Figure 1 shows anthocyanin content (mg/L) and total polyphenol index (TPI) (calculated by ITV method) of four different cabernet sauvignon plots (ITCS41, ITCS42, ITCS51, ITCS52) as a function of time, over four years of study (2008, 2009, 2010, and 2011). The anthocyanin content (mg/L) increases to reach a peak (optimal harvest time) and then decreases. This kinetic was observed in many previous studies [12,13,14,15]. The decrease of anthocyanins after the peak can be due to the combination of anthocyanins and tannins [16]. For the same year, the peak of anthocyanins is almost reached at the same date for all the plots. For example, the optimal harvest time of ITCS41, ITCS42, ITCS51 and ITCS52 was 29 September 2011. Moreover, all the concentrations of anthocyanins (at harvest time) in 2008 and 2010 are higher than those obtained in 2009 and 2011. The values of TPI increase in the beginning of grape maturation to reach a peak, then gradually decrease. This was observed for the four plots of CS and over the four years. Similarly to anthocyanin content, the levels of TPI in 2008 and 2010 are higher than those obtained in 2009 and 2011. Moreover, the TPI peaks were reached at the same time than those of anthocyanin content.

**Figure 2:**
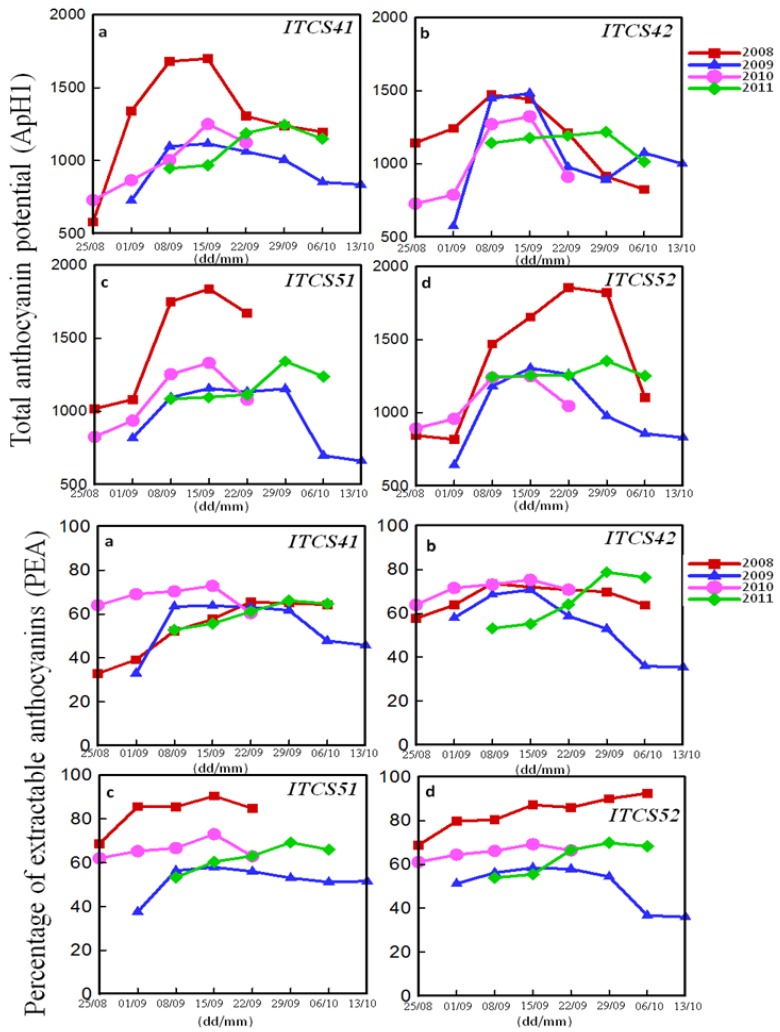
Total anthocyanin potential (AntpH1) and percentage of extractable anthocyanin (PEA) (calculated by Glories method) of four plots ITCS41 (**a**); ITCS42 (**b**); ITCS51 (**c**); and ITCS52 (**d**) of Cabernet Sauvignon grapes as a function of time (day (dd)/month (mm)) over four years (2008, 2009, 2010, and 2011).

Figure 2 shows, respectively, the total anthocyanin potential (AntpH1) and the percentage of extractable anthocyanins (PEA) (calculated by the Glories method) of four different cabernet sauvignon plots (ITCS41, ITCS42, ITCS51, ITCS52) as a function of time. The evolution of the Glories parameters (AntpH1 and PEA) is similar to those of ITV (anthocyanins and TPI). AntpH1 and PEA increase to a maximum value then decrease. The highest values were obtained in 2008 and 2010.

**Figure 3:**
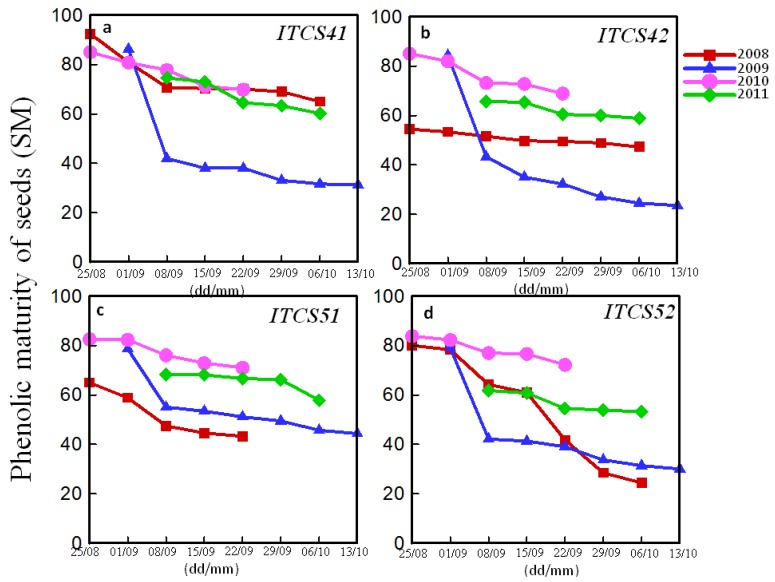
Seeds phenolic maturity of four plots ITCS41 (**a**); ITCS42 (**b**); ITCS51 (**c**); and ITCS52 (**d**) of Cabernet Sauvignon grapes as a function of time (day (dd)/month (mm)) over four years (2008, 2009, 2010, and 2011).

Figure 3 shows the evolution of seed maturity (SM) (calculated by the Glories method) as a function of time for different plots of CS. SM gradually decreases to reach a plateau. SM determines the role of seeds in the tannin content of the wine. The low values of SM are an indicator of high quality due to the higher proportion of skin tannins permitting the amplification of the extraction during grape maceration. Skin tannins are involved in the tannins-anthocyanins complex formation responsible for the wine color. A high amount of skin tannin, therefore, leads to a stable color [17].

### 3.2. Comparison between ITV and Glories Methods

#### 3.2.1. Phenolic Peaks

Table 1 shows the dates of the total polyphenol index (TPI) peaks obtained by the ITV method and the total phenolic richness (TPR) peaks obtained by the Glories method for all the plots of Cabernet Sauvignon (in 2008). The dates of the peaks for TPI and TPR are almost the same for all of the studied samples, even from different plots and domains (Supplementary Materials Table S1).

The same results were obtained for the years 2010 and 2011 and with other parameters of ITV and Glories, such as anthocyanin and AntpH1 peaks (Supplementary Materials Table S2). Given that the grape harvest dates were the same, a strong correlation therefore exists between the Glories and ITV methods. Both methods were shown to be adequate to measure grape phenolic maturity predicting some of the wine’s characteristics. High correlation coefficients were shown between anthocyanin content and TPI of harvested grapes with their corresponding produced wine [18].

#### 3.2.2. Correlation Matrix of ITV and Glories Parameters

The correlation matrix between the ITV and Glories parameters for Cabernet Sauvignon grapes over the four years (2008, 2009, 2010, and 2011) was also conducted (Supplementary Materials Table S3). A strong correlation was shown between the parameters (anthocyanins (ANT), total anthocyanin potential (TAP) and total polyphenol index (TPI)) obtained by the ITV method. (R^2^ = 0.7 – 0.93). For the Glories method, the correlation is strong between the total anthocyanins potential (AntpH1) and the potential of extractable anthocyanins (AntpH3.2). The ITV method allows the monitoring of phenolic maturity by the quantification of total phenolic compounds and anthocyanins. However, the quantitative evolution of anthocyanins in grapes is simultaneously done with that of tannins. The latter are quantified by the Glories method. Phenolic maturity of grapes is, therefore, optimized by the application of both ITV and Glories. The correlation between ITV and Glories was validated for *Vitis vinifera* L. grapes belonging to different varieties: Cabernet Sauvignon, Merlot, Syrah and Cabernet Franc. The interdependence between the technological and phenolic maturities was observed for the determination of harvest dates for the grapes of different varieties. However, the concentration in phenolic content was different between the different varieties.

### 3.3. Comparison between Technological and Phenolic Harvest Dates

Technological maturity can be determined by different indices such as titratable acidity and sugar content, whereas phenolic maturity takes into account anthocyanins, tannins and total phenolic concentration. The latter defines grape maturity and the phenolic compounds potential required in the future wine.

Table 2 shows technological harvest dates and phenolic peak for different plots of Cabernet Sauvignon in 2008. For all of the studied grape samples over 2008, 2009 and 2011 (**data not shown**), KACS31 and KACS32 plots for the KANAFAR domain; ITCS41 ITCS42, ITCS51 and ITCS52 plots for the ITANY domain, and MVCS51 and MVCSK0 plots for the MANSOURA domain present early phenolic peak dates compared to technological harvest dates. This implies that these plots have reached a full phenolic maturity before being harvested. Indeed, the technological harvest date is one week late compared to the phenolic peak. Moreover, Cabernet Sauvignon is a grape variety that must be harvested slightly overripe (about a week after the peak of anthocyanins) [15,19]. The plots of CS grapes were therefore harvested at an optimum date in 2008, 2009, and 2011 since both technological and phenolic maturities were reached. For the harvest in 2010 (Supplementary Materials Table S4) and for the majority of plots (MVCSK0, ITCS51, ITCS52, TACS6, KACS31, and KACS32), the phenolic harvest peak was slightly delayed compared to the technological harvest date. Over four years, the majority of Cabernet Sauvignon grapes presented a good phenolic ripeness when they were harvested.

### 3.4. Evolution of Climate for 2008, 2009, 2010 and 2011 Harvests

**Figure 4:**
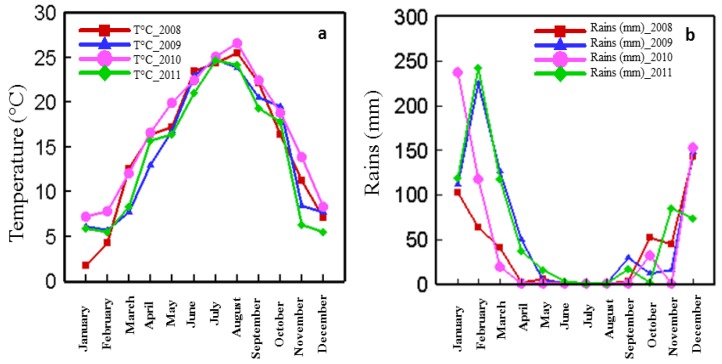
Temperature (**a**) and rainfall (**b**) as a function of time over four years (2008, 2009, 2010, and 2011).

The climate is an important factor that determines the quality of the wine [10] since the synthesis of phenolic compounds depends on it. More specifically, the quality of the wine is affected by the average annual precipitation and temperature [15]. Figure 4 shows the average annual amount of rainfall (mm) and temperature (°C) over the four studied years.

The evolution of temperature as a function of months is similar in 2008, 2009, 2010, and 2011. It gradually increases to reach a peak in August, then decreases. For the four Cabernet Sauvignon samples, phenolic and anthocyanin peaks were reached in September (Figure 1). Higher temperatures were observed during September 2008 and 2010 (T = 22.16 °C and 22.42 °C, respectively) compared to 2009 and 2011 (T = 20.55 °C and 19.37 °C, respectively). Similarly, the harvest dates of phenolic peaks for Cabernet Franc, Merlot, Syrah, and Petit Verdot had higher temperatures in 2008 and 2010, compared to 2009 and 2011. The highest temperatures observed in 2008 and 2010 were correlated to a higher phenolic and anthocyanin potentials of grapes. A warmer climate was shown to damage grape skins, thus improving anthocyanin and phenolic extractability [20]. Moreover, the accumulation of anthocyanins in grape skins was shown to be favored by high temperatures, thus affecting grape color [21]. The resulting wine is therefore rich in aromas, anthocyanins and phenolic compounds [10,22].

The evolution of the annual average precipitation as a function of months is similar in 2008, 2009, 2010, and 2011. It gradually decreases to reach null values in June, July and August, and then increases (Figure 4). During September, corresponding to the CS phenolic peaks, rainfall values were 0 mm, 3 mm, 16.5 mm, and 29 mm in 2010, 2008, 2011, and 2009 respectively. A higher phenolic potential was observed in the 2008 and 2010 harvest also for Cabernet Franc, Merlot, Syrah, and Petit Verdot varieties (**data not shown**), which were drier years than 2009 and 2011. Water stress was shown to increase the synthesis of phenolic compounds [23,24]. A restriction of water supply to the vines increases the quality potential of the harvests, especially for the production of red wines [23,24]. Moreover, anthocyanin peak dates in 2008 and 2010 were precocious for all grape varieties compared to 2009 and 2011. It has been shown that drought accelerates grape maturity [25].

## 4. Conclusions

Technological maturity of 642 samples was determined by the measurement of the titratable acidity and sugar content of the grapes. Glories and ITV methods were used to define their phenolic maturity. The correlation between ITV and Glories suggested that both methods are complementary to identify optimal harvest dates. Similarly, interdependence was observed between technological and phenolic maturity for the determination of harvest dates. The hottest and driest years enhanced phenolic compound quantities in grapes. All of the obtained results were validated for *Vitis vinifera* L. grapes belonging to different varieties: Cabernet Sauvignon, Merlot, Syrah, and Cabernet Franc.

## Figures and Tables

**Table 1:** Dates of peaks of the total polyphenol index and phenolic richness for each plot of Cabernet Sauvignon respectively detected by the ITV and Glories methods (1 and 2) for 2008 and 2009 harvests.

Domain	Plot	Codex	Peaks of Total Polyphenol Index (mg/L) (ITV Method)		Peaks of Phenolic Richness (RPT) (Glories Method)	
			**Harvest 2008**	**Harvest 2009**	**Harvest 2008**	**Harvest 2009**
**Itany**	**Cabernet 1994-1**	**ITCS41**	15-September	14-September	15-September	14-September
**Itany**	**Cabernet 1994-2**	**ITCS42**	8-September	14-September	8-September	14-September
**Itany**	**Cabernet 1995-1**	**ITCS51**	15-September	14-September	15-September	14-September
**Itany**	**Cabernet 1995-2**	**ITCS52**	15-September	14-September	22-September	14-September

**Table 2:** Technological harvest date and phenolic peak for each plot of Cabernet Sauvignon for the 2008 vintage.

Domain	Plot	Codex	Date of Harvest 2008	
			**Technological**	**Phenolic**
**Mansoura**	**Cabernet Y. HA. 1995-1**	**MVCS51**	22 to 25-September	15-September
**Mansoura**	**Cabernet K. CH. 1990**	**MVCSK0**	15 to 17-September	8-September
**Itany**	**Cabernet 1994-1**	**ITCS41**	18 to 20-September	15-September
**Itany**	**Cabernet 1994-2**	**ITCS42**	18-September	8-September
**Itany**	**Cabernet 1995-1**	**ITCS51**	20 to 24-September	15-September
**Itany**	**Cabernet 1995-2**	**ITCS52**	19 to 23-September	15-September
**Taanayel**	**Cabernet B6**	**TACS6**	25 to 10-October	29-September
**Kanafar**	**Cabernet 1**	**KACS31**	18 to 19-September	15-September
**Kanafar**	**Cabernet 2**	**KACS32**	18-September	15-September

## Supplementary Materials

The following are available online at www.mdpi.com/2076-3921/6/1/8/s1, Table S1. Page 7_line 16: Dates of peaks of total polyphenol index (TPI) and phenolic richness (RPT) for each plot of Cabernet Sauvignon respectively detected by the ITV and Glories methods (1 and 2) for 2008, 2009, 2010 and 2011 harvests. Table S2. Dates of peaks of anthocyanin (ANT) and the potential of easily extractable anthocyanins (AntpH3.2) for each plot of Cabernet Sauvignon respectively detected by the ITV and Glories methods (1 and 2) for 2008, 2009, 2010 and 2011 harvests. Table S3. Correlation matrix between ITV and Glories parameters for Cabernet Sauvignon grapes over four years (2008, 2009, 2010 and 2011). Table S4. Technological harvest date and phenolic peak for each plot of Cabernet Sauvignon for the 2008, 2009, 2010 and 2011 vintage.
