# Supplementary material for: A Comparative Study of the Phenolic and Technological Maturities of Red Grapes Grown in Lebanon
Source: Antioxidants (Basel). 2017 Jan 26;6(1):8. doi: 10.3390/antiox6010008 (PMC5383968; doi:10.3390/antiox6010008)
Supplement: Supplementary file 1 [file antioxidants-06-00008-s001.pdf]

Supplementary Materials: A Comparative Study of the Phenolic and Technological Maturities of Red Grapes Grown in Lebanon

Hiba N. Rajha, Nada El Darra, Sally El Kantar, Zeina Hobaika, Nicolas Louka and Richard G. Maroun

Table S1. Page 7_line 16: Dates of peaks of total polyphenol index (TPI) and phenolic richness (RPT) for each plot of Cabernet Sauvignon respectively detected by the ITV and Glories methods (1 and 2) for 2008, 2009, 2010 and 2011 harvests.

Domain	Plot	Codex	Peaks of total polyphenol index TPI (mg/L) (ITV method)				Peaks of phenolic richness (RPT) (Glories method)			
			**Harvest 2008**	**Harvest 2009**	**Harvest 2010**	**Harvest 2011**	**Harvest 2008**	**Harvest 2009**	**Harvest 2010**	**Harvest 2011**
MANSOURA	Cabernet Y. HA. 1995-1	MVCS51	15-Septembere	14-September	13-September	26-September	15-September	14-September	13-September	26-September
MANSOURA	Cabernet K. CH. 1990	MVCSK0	8-September	14-September	13-September	26-September	8-September	14-September	13-September	26-September
ITANY	Cabernet 1994-1	ITCS41	15-September	14-September	13-September	26-September	15-September	14-September	13-September	26-September
ITANY	Cabernet 1994-2	ITCS42	8-September	14-September	13-September	26-September	8-September	14-September	13-September	26-September
ITANY	Cabernet 1995-1	ITCS51	15-September	14-September	13-September	26-September	15-September	14-September	13-September	26-September
ITANY	Cabernet 1995-2	ITCS52	15-September	14-September	13-September	26-September	22-September	14-September	13-September	26-September
TANAYEL	Cabernet B6	TACS6	29-September	14-September	13-September	26-September	29-September	14-September	13-September	26-September
KANAFAR	Cabernet 1	KACS31	15-September	14-September	13-September	26-September	22-September	14-September	13-September	26-September
KANAFAR	Cabernet 2	KACS32	15-September	14-September	13-September	26-September	15-September	14-September	13-September	26-September

Table S2. Dates of peaks of anthocyanin (ANT) and the potential of easily extractable anthocyanins (AntpH3.2) for each plot of Cabernet Sauvignon respectively detected by the ITV and Glories methods (1 and 2) for 2008, 2009, 2010 and 2011 harvests.

Domain	Plot	Codex	Peaks of anthocyanins (ANT) (mg/L) (ITV method)				Peaks of potential of easily extractable anthocyanins (AntpH3.2) (Glories method)			
			Harvest 2008	Harvest 2009	Harvest 2010	Harvest 2011	Harvest 2008	Harvest 2009	Harvest 2010	Harvest 2011
MANSOURA	Cabernet Y. HA. 1995-1	MVCS51	15-September	14-September	13-September	26-September	15-September	14-September	13-September	26-September
MANSOURA	Cabernet K. CH. 1990	MVCSK0	8-September	14-September	13-September	26-September	8-September	14-September	13-September	26-September
ITANY	Cabernet 1994-1	ITCS41	15-September	14-September	13-September	26-September	15-September	14-September	13-September	26-September
ITANY	Cabernet 1994-2	ITCS42	8-September	14-September	13-September	26-September	8-September	14-September	13-September	26-September
ITANY	Cabernet 1995-1	ITCS51	15-September	14-September	13-September	26-September	15-September	14-September	13-September	26-September
ITANY	Cabernet 1995-2	ITCS52	15-September	14-September	13-September	26-September	22-September	14-September	13-September	26-September
TANAYEL	Cabernet B6	TACS6	29-September	14-September	13-September	26-September	29-September	14-September	13-September	26-September
KANAFAR	Cabernet 1	KACS31	15-September	14-September	13-September	26-September	22-September	14-September	13-September	26-September
KANAFAR	Cabernet 2	KACS32	15-September	14-September	13-September	26-September	15-September	14-September	13-September	26-September

Table S3. Correlation matrix between ITV and Glories parameters for Cabernet Sauvignon grapes over four years (2008, 2009, 2010 and 2011).

Correlation matrix		ITV			GLORIES			
	Parameters	ANT (mg/L)	TAP (mg/kg)	TPI	AntpH1 (mg/L)	AntpH3.2 mg/L	TPR	SP
ITV	ANT (mg/L)	1.00	0.93	0.83	0.84	0.74	0.35	0.20
	TAP (mg/kg)	0.93	1.00	0.69	0.87	0.60	0.20	0.32
	TPI	0.83	0.69	1.00	0.70	0.80	0.60	0.07
GLORIES	AntpH1 (mg/L)	0.84	0.87	0.70	1.00	0.70	0.30	0.25
	AntpH3.2 mg/L	0.74	0.60	0.80	0.70	1.00	0.70	0.08
	TPR	0.35	0.20	0.60	0.30	0.70	1.00	0.04
	SP	0.20	0.32	0.07	0.25	0.08	0.04	1.00

Table S4. Technological harvest date and phenolic peak for each plot of Cabernet Sauvignon for the 2008, 2009, 2010 and 2011 vintage.

Domain	Plot	Codex	Date of Harvest 2008		Date of Harvest 2009		Date of Harvest 2010		Date of Harvest 2011	
			Technological	Phenolic	Technological	Phenolic	Technological	Phenolic	Technological	Phenolic
**MANSOURA**	**Cabernet Y. HA. 1995-1**	**MVCS51**	22 to 25-September	15-September	28 to 30-September	14-September	23 to 25-September	13-September	21 to 22-September	26-September
**MANSOURA**	**Cabernet K. CH. 1990**	**MVCSK0**	15 to 17-September	8-September	5 to 7-October	14-September	7 to 8-September	13-September	4 to 5-October	26-September
**ITANY**	**Cabernet 1994-1**	**ITCS41**	18 to 20-September	15-September	13 to 17-October	14-September	13 to 14-September	13-September	13 to 14-October	26-September
**ITANY**	**Cabernet 1994-2**	**ITCS42**	18-September	8-September	14 to 16-October	14-September	9 to 14-September	13-September	3-October	26-September
**ITANY**	**Cabernet 1995-1**	**ITCS51**	20 to 24-September	15-September	7 to 10-October	14-September	7 to 8-September	13-September	10 to 11-October	26-September
**ITANY**	**Cabernet 1995-2**	**ITCS52**	19 to 23-September	15-September	9 to 14-October	14-September	8 to 9-September	13-September	4 to 15-October	26-September
**TANAYEL**	**Cabernet B6**	**TACS6**	25 to 10-October	29-September	9 to 10-October	14-September	6 to 8-September	13-September	3 to 10-October	26-September
**KANAFAR**	**Cabernet 1**	**KACS31**	18 to 19-September	15-September	12-October	14-September	11-September	13-September	14-October	26-September
**KANAFAR**	**Cabernet 2**	**KACS32**	18-September	15-September	13-October	14-September	11-September	13-September	15-September	26-September

© 2017 by the authors; licensee MDPI, Basel, Switzerland. This article is an open access article distributed under the terms and conditions of the Creative Commons Attribution (CC BY) license (http://creativecommons.org/licenses/by/4.0/).
